# Intraoral perfusion assessment using endoscopic hyperspectral imaging (EHSI)– first description of a novel approach

**DOI:** 10.1007/s00784-025-06197-5

**Published:** 2025-02-05

**Authors:** Paul Römer, Sebastian Blatt, Fabia Siegberg, Shankeeth Vinayahalingam, Bilal Al-Nawas, P. W. Kämmerer, Daniel G. E. Thiem

**Affiliations:** 1 Department of Oral and Maxillofacial Surgery, Facial Plastic Surgery, University Medical Centre Mainz, Augustusplatz 2, 55131 Mainz, Germany; 2 Department of Oral and Maxillofacial Surgery, Radboud University Medical Center, Nijmegen, The Netherlands

**Keywords:** Endoscopic HSI, Hyperspectral imaging, Intraoral perfusion monitoring, Intraligamentary anesthesia, Mucosal perfusion, Ischemia

## Abstract

**Objectives:**

This study aimed to establish a method to detect and quantify mucosal malperfusion intraorally using state-of-the-art Endoscopic Hyperspectral Imaging (EHSI). For this purpose, mucosal ischemia was selectively induced by intraligamentary anesthesia (ILA) with and without + epinephrine using a standardized protocol.

**Materials and methods:**

EHSI was performed using a novel endoscopic hyperspectral imaging system. Parameters assessed were Tissue Oxygen Saturation (StO_2_ [%]), Tissue Hemoglobin Index (THI), Near Infrared Perfusion Index (NPI) and Tissue Water Index (TWI). Fifty-seven healthy subjects received ILA using Articaine 4% with (ILA+) and without (ILA-) epinephrine at a dosage of 1:200,000 administered mesially and distally to the target tooth 42 (Universal No. 26). Mucosal perfusion was assessed using EHSI for 45 min post-injection.

**Results:**

After ILA+, a distinct ischemia of the mucosa was already clinically apparent after 30 s with significant reduction of THI and StO_2_ by an average of 57% (*p* < 0.001) and 7% (*p* < 0.040) compared to baseline values. Persistent hypoperfusion of the oral mucosa was observed throughout the monitoring period, exhibiting a gradual resolution at the 30-minute mark, and nearing baseline perfusion approximately 45 min post-injection. There was no papillary necrosis after ILA + injection.

**Conclusion:**

EHSI is suitable to adequately detect and visualize actual perfusion of the intraoral mucosa. The study revealed that LA with epinephrine (1:200,000) induce temporary hypoxia in the dental papilla but without causing severe ischemia.

**Clinical relevance:**

EHSI will enable promising applications in the future, i.a. success monitoring of periodontal therapies, intraoral free flap monitoring and the assessment of cancer margins.

## Introduction

Perfusion characteristics can provide insights into disease states or their dynamic changes in numerous clinical scenarios. In oral and maxillofacial surgery, dentistry, and otorhinolaryngology, the oral cavity and oropharynx are the sites of various diseases, commonly manifesting at the gingiva and the surrounding mucosal linings. These include inflammatory diseases such as gingivitis, periodontitis and aphthous lesions, but also premalignant lesions like leukoplakia, erythroplakia, oral lichen planus or mucosal dysplasia. Endoscopic hyperspectral imaging (eHSI) is an advanced optical imaging technique that captures and analyzes a wide spectrum of light across multiple wavelengths beyond the visible spectrum. By measuring the interaction of light with biological tissues, eHSI generates detailed spectral data that reflect tissue composition, oxygenation, and perfusion. This technique combines spatial and spectral information, allowing for non-invasive, real-time visualization and quantification of physiological and pathological processes. Using conventional hyperspectral imaging, the research team has previously been able to demonstrate that various types of tissue can be differentiated based on their characteristic absorption and reflection profiles [1]. Especially regarding potentially malignant oral mucosal lesions and the detection of resection margins as part of tumor surgery, hyperspectral imaging offers a promising approach for non-invasive diagnostics in the future. Furthermore, hyperspectral imaging also proved to be a reliable tool for monitoring microvascular transplants [2, 3]. However, the use of an endoscopic HSI procedure (eHSI) to detect intraoral mucosal perfusion is not studied. In this study measurement of mucosal blood flow after intraligamentary anesthesia was chosen to validate this new approach in a standardized measurement procedure. Intraligamentary anesthesia was chosen for this study due to its well-documented propensity to induce clinically observable, transient tissue ischemia. This effect arises from increased tissue pressure within the confined space of the periodontal ligament, resulting in a more reliable method for inducing localized ischemia compared to conventional infiltration anesthesia. Sufficient analgesia is a fundamental requirement for effective dental treatment. In addition to most commonly used techniques of classical infiltration and nerve block anesthesia, direct methods such as intraligamentary anesthesia (ILA) are available, which can provide selective analgesia of an individual tooth without the obligation to anesthetize an entire quadrant of teeth and their surrounding soft tissue [4]. Therefore, the anesthetic solution is slowly injected under high pressure directly into the periodontal space of the tooth by using special syringe systems, penetrating through the entire periodontium and the cancellous bone to the apex of the root and the adjacent tooth nerve, thus merely anesthetizing the targeted tooth [5, 6]. Many agents used today are combined with a vasoconstrictor, predominantly epinephrine, to enhance and prolong the anesthetic, respective ischemic effects [7]. However, because of some dose-dependent limitations due to potential cardiovascular and local toxic effects [8], a minimal possible use of vasoconstrictor should be considered [9, 10]. To prevent necrosis of the dental papillae due to excessive ischemic effects, the use of anesthetics with a concentration of epinephrine not higher than 1:200.000 is recommended for ILA. The aim of this study was to validate eHSI through the assessment and quantification of oral mucosal perfusion changes induced by intraligamentary anesthesia with and without adjunctive vasoconstrictors (Epinephrine 1:200,000).

## Materials and methods

### Study cohort

A total of 57 adult volunteers participated in this prospective, randomized clinical trial at the Department of Oral and Maxillofacial Surgery, Facial Plastic Surgery, University Medical Centre Mainz, Germany. The experiments took place as part of student teaching, which facilitated uniform application and documentation. All subjects consented in advance to the procedure and data collection. This study was approved by the local ethics committee of Rhineland-Palate (registration number: 2024–17582) and was conducted in accordance with the protocol and in compliance with the moral, ethical, and scientific principles governing clinical research set out in the Declaration of Helsinki of 1975 and revised in 1983. Thirty-six female and twenty-one male participants between 19 and 34 years of age were included. All subjects were healthy and had no cardiovascular risks, allergies nor known or suspected intolerances to local anesthetics and particularly not their individual chemical components. Patients with corresponding comorbidities, in particular patients with generalized inflammatory diseases of the periodontium or malignancies, were excluded from the study. A randomization of the participants into two groups was performed. One group received ILA with Articaine 4% containing Epinephrine at a dose of 1:200,000 (ILA+), whereas the control group received Articaine 4% without epinephrine (ILA-).

### Test substances and intraligamentary application

**Fig. 1:**
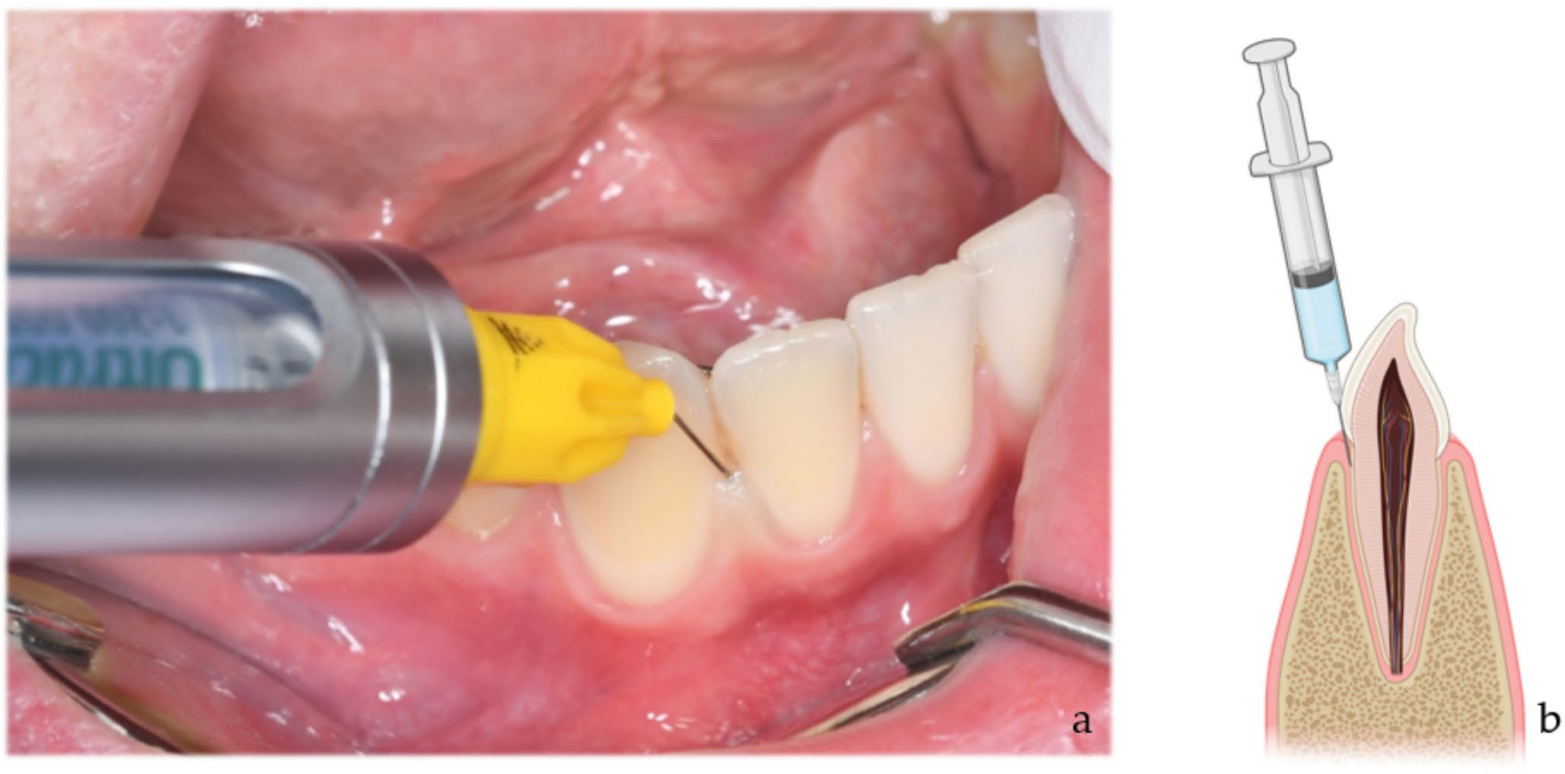
(**a**) Application of intraligamentary anesthesia with Articaine 4% on tooth 42, (**b**) schematic illustration

The experiments were conducted on a regular dental treatment unit at an ambient temperature of 22 °C. Each participant in the study group underwent one mesial and one distal intraligamentary injection with 0.2 mL of Articaine-4% + epinephrine 1:200,000 (40 mg/mL Articaine + 0.006 mg/mL epinephrine hydrochloride; Ultracain^®^D-S, Sanofi-aventis, Paris, France) (ILA+) on tooth number 42 each. ILA administration was performed lege artis with a special syringe system (SOPIRA^®^ Citoject N, Kulzer GmbH, Hanau, Germany) and a thin injection needle measuring 0.3 mm in diameter (SOPIRA Carpule^®^ Dentalnadeln, Kulzer GmbH, Hanau, Germany). The needle was inserted through the gingival sulcus at an angle of 10–20° to the longitudinal axis of the anterior tooth number 42 and extended 2 to 3 mm into the periodontal ligament between the root and the alveolar bone (Fig. 1). Subsequently, patient injection of 0.2 mL of the respective anesthetic was performed over a minimum of 20 s until counterpressure was achieved, followed by retention of the syringe in the sulcus for an additional 20 s. In the control group, a similar protocol was followed using Articaine 4% without epinephrine (ILA-).

### Hyperspectral imaging (HSI) and data acquisition

**Fig. 2:**
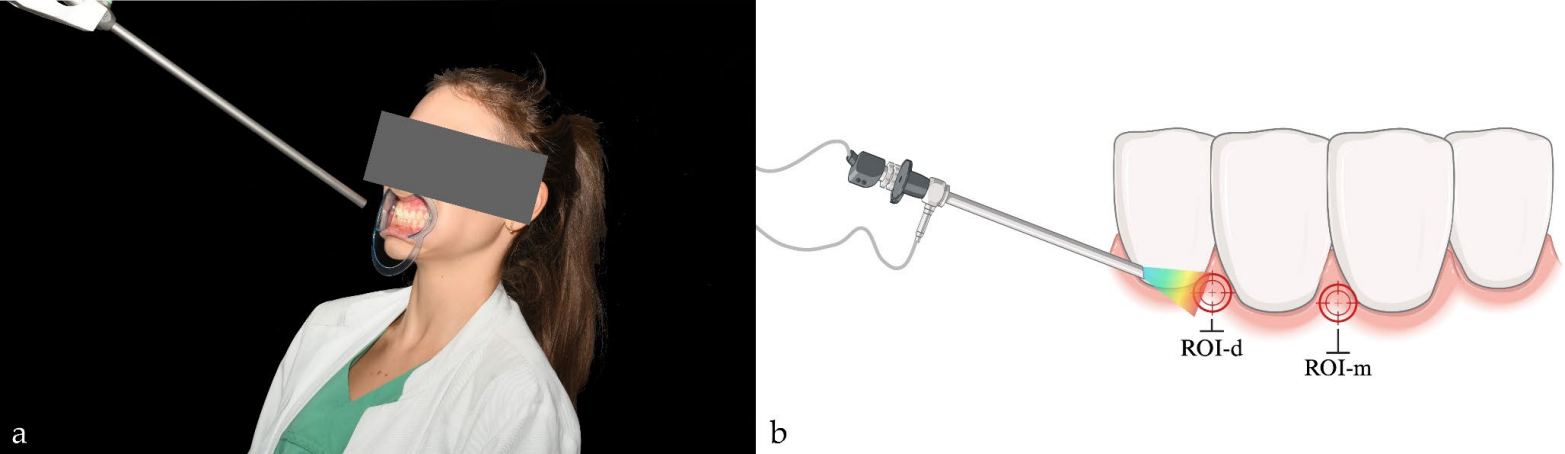
(**a**) Experimental setup and hyperspectral scan with focus on region 42 after local anesthesia, (**b**) schematic illustration (created with Biorender)

**Fig. 3:**
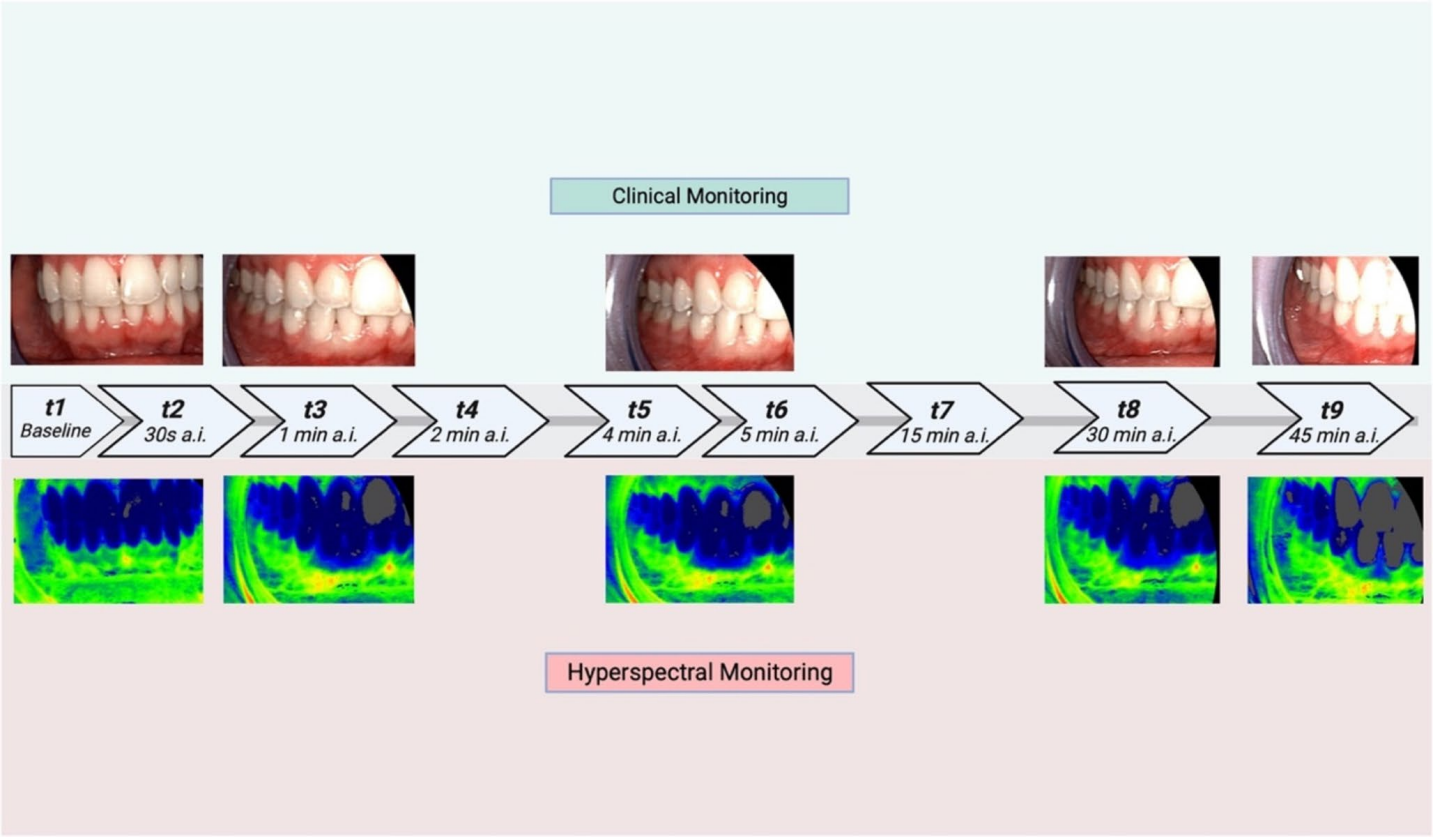
Measurement sequence between 0 and 45 min of THI with clinical ischemia after ILA+ (**top**: THI as false-color image, **bottom**: RGB image)

The hyperspectral image data sets were acquired using a state-of-the-art endoscopic hyperspectral sensor system (TIVITA Mini camera system^®^, Diaspective Vision GmbH, Am Salzhaff, Pepelow, Germany) capable of detecting 100 wavelengths in the range of 500 to 1000 nm, providing a bandwidth of 5 nm [11]. The system operates based on the so-called pushbroom spectrometer principle, allowing to detect physicochemical information of an object transferred with the remitted light spectrum. The light is transmitted through the lens to the optical system of the spectrometer, where it is first collimated and then split up into individual wavelengths by a transmission grating before reaching the sensor of the CMOS camera through the second optical system. Due to the design of the spectrometer unit, the spatial direction (width of the object; Y-axis) is directly detected, whereas the second spatial direction (length of the object; X-axis) results from continuous movement of the entry gap in the scan unit. Thus, the object is scanned lengthwise within a few seconds. A third, spectral dimension is determined using the recorded wavelengths, resulting in a 3D data cube (*X*, spatial dimension; *Y*, spatial dimension; *λ*, spectral dimension) [12]. Each pixel in the acquired data cube is assigned a full tissue spectrum in the above-mentioned wavelength range, allowing the generation of a three-dimensional Hypercube (HSI cube) - the prerequisite for analysis of tissue-specific chemical information. These physiological parameters calculated from the HSI cube include tissue-oxygen-saturation / superficial perfusion in percent [StO_2_ (0-100%)], perfusion in deeper tissue layers / Near-Infrared-Range as dimensionless value [NPI (0-100)], distribution of hemoglobin / Tissue-Hemoglobin-Index [THI (0-100)], tissue water content [TWI (0-100)] and fat content [TFI (0-100)], which can be displayed in RGB (red, green and blue) and false color images. According to the manufacturer, the TIVITA^®^ Tissue system does not require user calibration prior to each use, as it is pre-calibrated during manufacturing process. The calibration data are permanently stored within the camera, ensuring consistent performance and eliminating the need for additional on-site adjustments. Hyperspectral image acquisition on the subjects was performed at an angle of 90° between the targeted tooth and the endoscopic camera. A distance between 8 and 10 cm was maintained in order to generate regularly exposed and sharp scans. Cheek retractors were used for uniform examination conditions (Fig. 2). Image analysis and evaluation of the raw data was accomplished with the camera-specific software package (TIVITA^®^ Suite Mini). As regions of interest (ROIs), respectively, the mesial (ROI-m) and distal (ROI-d) interdental papilla of tooth 42 were utilized. Circular ROIs with a diameter of 15 pixels, corresponding to 5.29 mm, respectively an area of ~ 177 pixels were used to measure dynamic perfusion parameters. The image size was 720 × 540 pixels with an image resolution of 72 pixels per inch. The first eHSI recording was taken before local anesthesia injection to obtain baseline values. Further recordings followed the predetermined measurement protocol at specific time points after injection (a.i.) at t1 = Baseline, t2 = 30s, t3 = 1 min, t4 = 2 min, t5 = 4 min, t6 = 5 min, t7 = 15 min, t8 = 30 min and t9 = 45 min (Fig. 3).

### Statistics

The Null Hypothesis states that changes in oral mucosa perfusion cannot be reproducibly discriminated using endoscopic hyperspectral measurement. With a presumed effect size of 0.65 according to Cohen and a permissible α error = 0.05 at a targeted test power of 95%, the necessary number of cases was *n* = 53,74. Congruent to this calculation, a number of *n* = 57 subjects were recruited to conduct the study. The raw datasets collected were entered in Excel^®^ sheets (Microsoft Corporation, Redmond WA, USA) and subsequently transferred into SPSS-statistics^®^ (version 27 MacOS X; SPSS Inc., IBM; Corporation, Armonk, NY, USA). The acquired data were expressed as mean values (m) ± standard deviation (SD±), minimum (min), and maximum (max). A non-parametric Shapiro-Wilk-test^(+)^ was used to check for normal distribution. To account influences like various baseline values, rigorous statistical techniques were employed, including the Greenhouse-Geisser correction for violations of sphericity in repeated measures ANOVA to obtain representative results. Additionally, a correlation analysis was conducted to evaluate the strength and direction of associations between clinically observed ischemia and hyperspectral parameters. Data were analyzed for statistical significance using single factor variance analysis with repeated measures (ANOVA^(#)^), Student’s t-test^(*)^, Wilcoxon signed ranks test^(§)^, and unpaired non-parametric Mann-Whitney U test^($)^. In the absence of sphericity according to Mauchly W-test (*P* < 0.05), Greenhouse-Geisser correction^(§*)^ was applied in the case of epsilon < 0.75. Furthermore, to determine the association of clinical apparitional ischemia and hyperspectral parameters, we conducted a correlation analysis using Eta coefficient for nominal and metric scale levels. In this context, the Eta correlation coefficient (η) indexes the strength and direction of the relation between the two variables ischemia and the corresponding HSI-parameters. The partial eta squared (η_p_^2^) indicates the percentage of the statistically significant variation that can be explained by the change in the hyperspectral parameters StO_2_, NPI, THI, and TWI. An eta coefficient of|η| < 0.30 was considered a weak correlation, 0.30 ≤|η| < 0.50 a moderate correlation, and|η| ≥ 0.50 a strong correlation [13]. P-values ≤ 0.05 were considered significant. Histograms and line charts were used for illustration purposes.

## Results

### eHSI assessment of tissue perfusion, oxygenation, and water content

#### Baseline (t1)

**Fig. 4:**
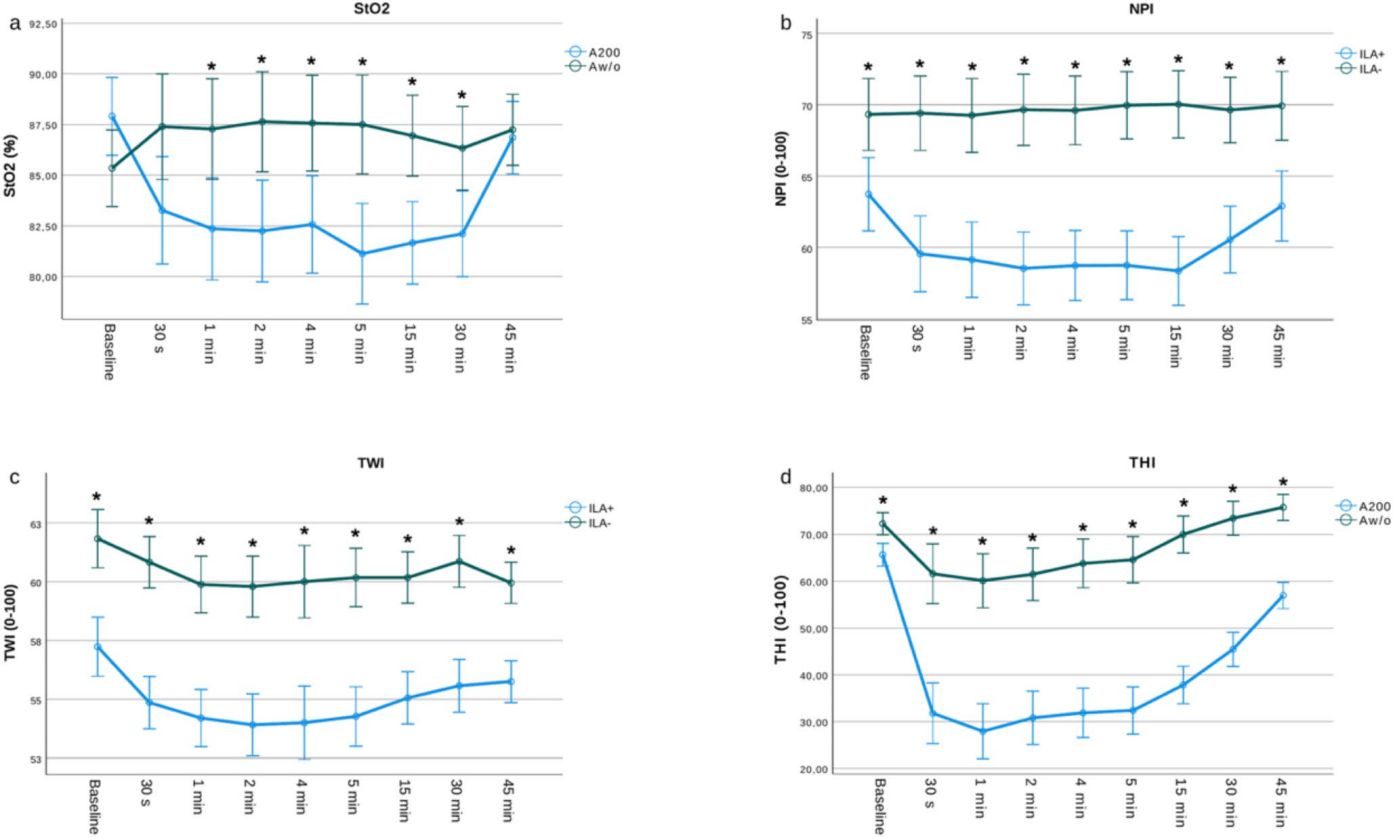
Line graphs demonstrate the group-specific course of StO2 (**A**), NPI (**B**), TWI (**C**) and THI (**D**) from Baseline to 45 min after ILA as mean values ± SD

At baseline, no significant differences were detected for StO_2_ between ILA + and ILA- (*P* = 0.082^($)^; mean: 86.6% ± 5.2; Fig. 4A). Before injection, baseline *NPI* showed significant differences (*P* < 0.001^($)^; mean ILA-: 69.3% ± 7.3; mean ILA+: 63.7% ± 6.2; Fig. 4B). TWI also revealed significant different between ILA + and ILA- (*P* < 0.001^($)^; mean ILA-: 61.8% ± 4.1; mean ILA+: 57.2% ± 2.2) (Fig. 4C; Table 1). THI measurements showed significant differences between ILA + and ILA- (*P* < 0.001^($)^; mean ILA-: 72.3% ± 6.464; mean ILA+: 65.6% ± 6.3; Fig. 4D).

**Table 1:** Analysis of eHSI parameters (StO2, NPI, TWI, and THI) at time point t1 presenting mean values and standard deviations (SD±) comparing ILA + and ILA- formulations

	eHSI	Formulation	Mean (%)	SD±	Mann-Whitney-test (*P*-value)
t1	StO2 (%)	ILA+	87.91	4.54	0.082
		ILA-	85.35	5.54	
	NPI	ILA+	63.73	6.2	< 0.001
		ILA-	69.33	7.31	
	TWI	ILA+	57.23	2.19	< 0.001
		ILA-	61.83	4.12	
	THI	ILA+	65.64	6.31	< 0.001
		ILA-	72.28	6.46	

#### 30 seconds a.i. (t2)

There were no significant differences in StO_2_ perfusion thirty seconds after injection between both groups (*P* = 0.064^($)^; mean: 85.7% ± 7.2). However, in the ILA- group, a reflective hyperemia with a minor increase in StO_2_ could be observed. THI showed a clear drop and significant reduction in tissue hemoglobin concentration between both substance groups thirty seconds a.i. (*P* < 0.001^($)^; mean ILA-: 61.6% ± 19.4; mean ILA+: 31.8% ± 14.4). Thirty seconds after injection, NPI revealed significantly decreased in ILA + compared to ILA- (*P* < 0.001^($)^; mean ILA-: 69.4% ± 7.1; mean ILA+: 59.6% ± 6.9). Tissue Water Index (TWI) differed significantly between ILA + and ILA- (*P* < 0.001^($)^; ILA-: 60.8% ± 3.1; ILA+: 54.9% ± 2.7) (Table 2).

**Table 2:** Analysis of eHSI parameters (StO2, NPI, TWI, and THI) at time point t2 presenting mean values and standard deviations (SD±) comparing ILA + and ILA- formulations

	eHSI	Formulation	Mean (%)	SD±	Mann-Whitney-test (*P*-value)
t2	StO2 (%)	ILA+	83.27	8.28	0.064
		ILA-	87.4	5.47	
	NPI	ILA+	59.55	6.98	< 0.001
		ILA-	69.41	7.06	
	TWI	ILA+	54.86	2.71	< 0.001
		ILA-	60.83	3.15	
	THI	ILA+	31.82	14.36	< 0.001
		ILA-	61.59	19.41	

#### 1 minute a.i. (t3)

After one minute, significant differences in StO_2_ perfusion were observed (*P* = 0.009^($)^; ILA-: 87.3% ± 5.6; ILA+: 82.4% ± 7.6). For ILA + THI, NPI, and TWI were significantly lower compared to ILA- (*P* < 0.001^($)^ in each case) (Table 3).

**Table 3:** Analysis of eHSI parameters (StO2, NPI, TWI, and THI) at time point t3 presenting mean values and standard deviations (SD±) comparing ILA + and ILA- formulations

	eHSI	Formulation	Mean (%)	SD±	Mann-Whitney-test (*P*-value)
t3	StO2 (%)	ILA+	82.36	7.64	0.009
		ILA-	87.28	5.56	
	NPI	ILA+	59.14	6.69	< 0.001
		ILA-	69.26	7.24	
	TWI	ILA+	54.2	3.37	< 0.001
		ILA-	59.88	3.07	
	THI	ILA+	27.98	11.54	< 0.001
		ILA-	60.1	18.55	

#### 2 and 4 minutes a.i. (t4,5)

At two and four minutes after injection, a significant reduction in StO_2_ was evident in the ILA + group compared to ILA- (*P* = 0.005^($)^ in each case). The group with added epinephrine (ILA+) also showed a significant reduction in THI, NPI and TWI at two and four minutes a.i. (*P* < 0.001^($)^; Table 4)

**Table 4:** Analysis of eHSI parameters (StO2, NPI, TWI, and THI) at time points t4 and t5 presenting mean values and standard deviations (SD±) comparing ILA + and ILA- formulations

	eHSI	Formulation	Mean (%)	SD±	Mann-Whitney-test (*P*-value)
t4	StO2 (%)	ILA+	82.25	7.72	0.005
		ILA-	87.64	5.41	
	NPI	ILA+	58.54	6.68	< 0.001
		ILA-	69.66	6.75	
	TWI	ILA+	53.91	3.6	< 0.001
		ILA-	59.79	3.37	
	THI	ILA+	30.84	12.32	< 0.001
		ILA-	61.48	17.26	
t5	StO2 (%)	ILA+	82.57	7.51	0.05
		ILA-	87.57	4.95	
	NPI	ILA+	58.73	5.99	< 0.001
		ILA-	69.6	6.9	
	TWI	ILA+	54.0	4.72	< 0.001
		ILA-	60.0	3.48	
	THI	ILA+	31.91	11.97	< 0.001
		ILA-	63.79	15.7	

#### 5 and 15 minutes a.i. (t6,7)

After five and 15 min, StO_2_ perfusion in the ILA + group continued to show significantly lower levels compared with ILA- (*P* = 0.002^($)^). There was a significant reduction of THI, NPI and TWI for ILA + and ILA- at t6 and t7 (*P* < 0.001^($)^). However, after 15 min there was a slow upward trend for all parameters (StO_2_, NPI, TWI, THI) in the ILA + group (Table 5).

**Table 5:** Analysis of eHSI parameters (StO2, NPI, TWI, and THI) at time points t6 and t7 presenting mean values and standard deviations (SD±) comparing ILA + and ILA- formulations

	eHSI	Formulation	Mean	SD±	Mann-Whitney-test (*P*-value)
t6	StO2 (%)	ILA+	81.13	7.81	0.005
		ILA-	87.5	5.08	
	NPI	ILA+	58.75	5.61	< 0.001
		ILA-	69.97	6.96	
	TWI	ILA+	54.27	3.27	< 0.001
		ILA-	60.17	3.4	
	THI	ILA+	32.43	11.51	< 0.001
		ILA-	64.57	14.83	
t7	**StO2 (%)**	ILA+	81.66	5.67	0.005
		ILA-	86.95	5.07	
	NPI	ILA+	58.34	5.39	< 0.001
		ILA-	70.03	7.1	
	TWI	ILA+	55.05	3.36	< 0.001
		ILA-	60.17	2.46	
	THI	ILA+	37.84	10.66	< 0.001
		ILA-	69.98	10.52	

#### 30 minutes a.i. (t8)

At 30 min, StO_2_ revealed significantly lower for ILA + compared with ILA- (*P* = 0.013^($)^; ILA+: 82.1% ± 6.4 and ILA-: 86.3% ± 4.6). THI, NPI and TWI also increased in ILA + but were still significantly lower compared with ILA- (*P* < 0.001^($)^ in each case; Table 6).

**Table 6:** Analysis of eHSI parameters (StO2, NPI, TWI, and THI) at time point t8 presenting mean values and standard deviations (SD±) comparing ILA + and ILA- formulations

	eHSI	Formulation	Mean	SD±	Mann-Whitney-test (*P*-value)
t8	StO2 (%)	ILA+	82.11	6.42	0.013
		ILA-	86.33	4.58	
	NPI	ILA+	60.55	5.25	< 0.001
		ILA-	69.64	6.94	
	TWI	ILA+	55.57	2.89	< 0.001
		ILA-	60.86	3.04	
	THI	ILA+	45.48	10.1	< 0.001
		ILA-	73.43	9.26	

#### 45 minutes a.i. (t9)

At 45 min after injection, StO_2_ levels between the groups nearly converged, attributed to a marked increase in the ILA + group (*P* = 0.811^(§)^; ILA+: 86.9% ± 4.2 and ILA-: 87.2 ± 5.2). THI, NPI and TWI remained significantly lower in ILA + compared to ILA- (each *P* < 0.001^($)^; Table 7).

**Table 7:** Analysis of eHSI parameters (StO2, NPI, TWI, and THI) at time point t9 presenting mean values and standard deviations (SD±) comparing ILA + and ILA- formulations

	eHSI	Formulation	Mean (%)	SD±	Mann-Whitney-test (*P*-value)
t9	StO2 (%)	ILA+	86.86	4.21	0.811
		ILA-	87.24	5.17	
	NPI	ILA+	62.91	5.24	< 0.001
		ILA-	69.93	7.47	
	TWI	ILA+	55.75	2.61	< 0.001
		ILA-	59.95	2.08	
	THI	ILA+	56.96	6.77	< 0.001
		ILA-	75.76	7.99	

### Difference to baseline perfusion pattern

#### StO_2_

Overall, there were significant differences in mean StO_2_ in the ILA + from t3 (1 min) to t8 (30 min) a.i. compared to baseline (t3 *P* = 0.009^(#)^; t4 *P* = 0.002^(#)^; t5 *P* = 0.004^(#)^; t6 - t8 *P* < 0.001^(#)^*)*. At t2 (30 s a.i. and t9 (45 min a.i.), no significant differences were observed compared to StO_2_ baseline (t2 *P* = 0.131^(#)^; t9 *P* = 1.000^(#)^). In the ILA group, StO_2_ showed no significant difference when compared to the baseline at the various measurement time points (t2 - t3 *P =* 1.000^(#)^; *t4 P =* 0.517^(#)^; *t5 P =* 0.411^(#)^; t6 *P* = 0.864^(#)^; t7-t9 *P* = 1.000^(#)^) (Fig. 4A).

#### THI

For ILA+, THI significantly decreased over time when compared to t1 (baseline) (t2 to t8 *P* < 0.001^(#)^ and *t9 P* = 0.003^(#)^). In contrast, THI demonstrated a marginal decline from baseline over time (t2 *P* = 0.162^(#)^; t4 *P* = 0.056^(#)^; t5 *P* = 0.142^(#)^; t6 *P* = 0.136^(#)^; t7-t8 *P* = 1.000^(#)^; t9 *P* = 0.192^(#)^), whereby the most notable reduction was observed at t3 (1 min a.i.) with *P* = 0.042^(#)^ (Fig. 4D).

### Clinical monitoring

**Fig. 5:**
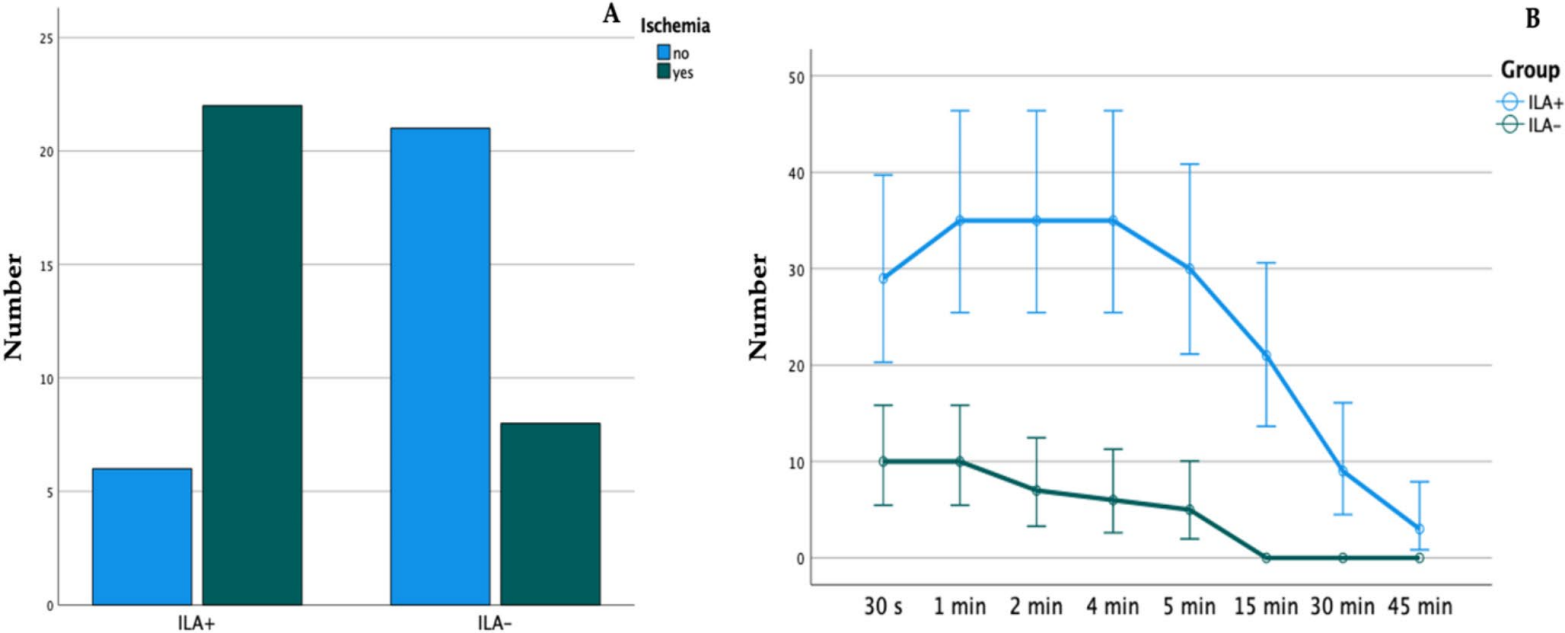
Number of cases with clinically detectable ischemia of the gingiva (**A**) and their progression over time referring to groups ILA + and ILA- (**B**)

Overall, after injection of ILA+, clinical ischemia of the gingiva occurred in 22 of 28 participants (78.6%) during the test period, whereas in the control group ILA-, ischemia was observed in only 8 of 29 cases (27.6%; *P* < 0.001^($)^). Furthermore, ischemia induced by ILA + was clearly prolonged over time (Fig. 5B). In this group, signs of ischemia were present during the entire measurement period, whereas in the control group (ILA-) no clinical signs of ischemia persisted after 15 min. In addition, no case of papillary necrosis was observed in the present study population. To further investigate the correlation between mucosal ischemia and hyperspectral imaging, a correlation analysis was performed.

#### Correlation between ischemia and StO_2_

A statistically moderate correlation was found between the occurrence of ischemia and StO_2_ (η = 0.454; *P* < 0.001^(#)^). With an eta squared of (η_p_^2^ = 0.206), only 20.6% of clinical ischemia can be explained based on the hyperspectral parameter StO_2_.

#### Correlation between ischemia and THI

For the Tissue Hemoglobin Index, a correlation coefficient of η = 0.7 (*P* < 0.001^(#)^) and thus a strong correlation with the occurrence of clinical ischemia could be determined. Corresponding to a η_p_^2^ of 0.5, changes in THI can statistically explain induced ischemia in 50.7% of the cases.

#### Correlation between ischemia and NPI

NPI indicated a low-moderate correlation at η = 0.374 (*P* < 0.001^(#)^). Therefore, only 14.0% of NPI value changes can be attributed to induced ischemia (η_p_^2^ = 0.140).

#### Correlation between ischemia and TWI

Only a weak correlation existed between ischemic gingiva and the Tissue Water Index (η = 0.204); *P* < 0.001^(#)^). Consequently, according to an η_p_^2^ of 0.041, ischemia can only be explained by TWI in 4.1% of cases.

## Discussion

This is the first study to investigate the intraoral use of a modern endoscopic hyperspectral sensor system (eHSI) by assessing dynamic perfusion changes of the oral mucosa and dental papillae after intraligamentary injection of local anesthesia with and without epinephrine (ILA + and ILA-). (e)HSI is a non-contact, non-invasive and radiation-free technique successfully used in medicine and other specialties, which combines conventional and spectroscopic methods to obtain both spatial and (hyper-) spectral image information detecting wavelengths from the visually visible spectrum (VIS; 350–740 nm) to Near-Infrared Perfusion-Index (NPI 750–1000 nm) [14, 15]. This technique enables rapid acquisition and immediate analysis of diagnostically relevant information, including determination of Oxyhemoglobin (O_2_HB) and Deoxyhemoglobin (HHb), thus allowing quantification of local tissue oxygenation [14]. Typical areas of application of eHSI include wound care and healing progress surveillance [14], as well as oncology for intraoperative tissue detection and determination of tumor margins [16, 17]. Furthermore, eHSI is utilized in reconstructive and vascular surgery for perioperative flap perfusion monitoring [2, 3], dermatology for burn and skin cancer assessment, as well as neurology and ophthalmology for real-time analysis of tissue perfusion. Compared to other sensors, such as “Oxygen to see” (O2C) [18] or Laser speckle contrast imaging (LSCI) [19], eHSI offers advantages through its user-friendly ability to capture and display large, high-resolution tissue areas in a single scan, providing RGB, spatial and spectral data without the need for direct contact [20]. Dental ultrasound (US) also represents a promising alternative for visualization of oral mucosal tissue. Advancements in US technology, including Doppler imaging and contrast-enhanced US, have significantly enhanced the capability to evaluate local tissue perfusion. However, the application of US in dental settings is distinctly user-dependent and requires expensive, specialized transducers [21, 22]. In a standardized measurement protocol, this clinical trial demonstrated that vasoactive effects of epinephrine in ILA + and the clinical appearance of gingival ischemia can be tracked over time via eHSI demonstrated by a significant reduction of tissue hemoglobin index-parameters. As a secondary result, the study revealed that visually detectable mucosal ischemia after ILA was apparent in ILA + over a period of > 45 min, whereas the persistence of ischemia was limited to 15 min when Articaine-4% without epinephrine (ILA-) was used (Fig. 5). Finally, a correlation analysis revealed that THI is the most reliable hyperspectral parameter for the intraoral monitoring of tissue ischemia.

Vasoconstrictors such as epinephrine contribute to successful and profound local anesthesia by especially improving duration of analgesia. However, various studies could not prove sufficient evidence that higher doses of epinephrine improve anesthetic efficacy [23–25]. Nevertheless, Moore et al. demonstrated in a study that the average blood loss during periodontal surgery was significantly lower using Articaine-4% + epinephrine at 1:100,000 compared with Articaine-4% + 1:200,000 epinephrine [26]. In comparison to infiltration anesthesia, intraligamentary anesthesia (ILA) does not predominantly aim to induce ischemia, while still ensuring adequate analgesic efficacy, due to the higher risk of papillary necrosis [27]. However, to date, there are no representative studies regarding the occurrence of pressure- or ischemia-induced necrosis of interdental papillae after intraligamentary anesthesia. In the present study, we were able to demonstrate that, based on the measured hyperspectral parameters, ILA with the addition of epinephrine 1:200,000 (ILA+) results in reduced blood flow in the surrounding mucosa (decreased THI) due to vasoconstriction; however, it does not induce hypoxemia of the remaining tissue hemoglobin (nearly constant StO2). Based on our results, ILA + caused a maximum reduction of THI by 57.3%, whereas StO2 only decreased by 7.7% compared with baseline values. After injection of ILA-, however, THI only decreased by 16,9% compared to baseline, while StO2 even increased by 2,7%. Furthermore, the present results revealed that when using Articaine-4% containing 1:200,000 epinephrine, its vasoactive effect progressively subsides after 30 min and returns to nearly 87% of the baseline (THI) after 45 min (Fig. 4). The perfusion changes observed after intraligamentary anesthesia with epinephrine align to its pharmacokinetics. Epinephrine has a plasma half-life of 2–3 min due to rapid degradation by catechol-O-methyltransferase (COMT) and monoamine oxidase (MAO). However, its local vasoconstrictive effects persist longer due to sustained binding to alpha-adrenergic receptors, reducing local blood flow at the injection site. In the present study, perfusion parameters like THI and StO2 showed significant reductions within minutes post-injection, leading to a peak reduction at 5 min and gradually returning to baseline values by 30–45 min. This chronological sequence reflects typical pharmacodynamics of epinephrine, where local effects outlast its systemic clearance. Similarly, tissue oxygenation saturation (StO_2_) revealed no significant difference between ILA + and ILA- after 45 min (Fig. 2). Consequently, with dosages maintained up to a ratio of 1:200,000, it is anticipated that the threshold for critical hypoxia will not be surpassed. This suggests a minimal likelihood of encountering vasoconstrictor-induced papillary necrosis under these conditions. Compared with own preliminary studies, the vasoactive effects of the local anesthetics applied occurred considerably earlier [15]. Thiem et al. demonstrated a maximum reduction of StO_2_ below baseline not earlier than 30 in ILA + and 120 min in ILA-, after cutaneous injection of Articaine 4% ± epinephrine 1:200,000 [15]. In the present study, such a significant drop in StO_2_ below baseline values occurred already after 30 s and reached its minimum after five minutes, but only in the ILA + group. The different results of both studies can most likely be attributed to the various modalities of the measurement sites and the fact that the injected drug can occupy significantly more anatomical space for diffusion when injected within the dermis compared to direct application into the much denser dental sulcus [28]. Moreover, the study by Thiem et al. focused exclusively on superficial skin perfusion (StO_2_), whereas according to the results of this study, changes in THI value particularly correlate with the occurrence of gingival ischemia after being evoked by intraligamentous injections. In both clinical trials, a reflective hyperemia was represented by an StO_2_ increase following epinephrine-free LA application [15]. There are currently no similar studies that have documented dynamic perfusion changes of the oral mucosa using HSI which impairs comparison with other studies. However, there are various efforts in the field of visceral surgery that have investigated the occurrence of necrosis and ischemia using hyperspectral imaging. For example, Felli et al. were able to demonstrate a significant reduction in StO_2_ and OHI (Organ Hemoglobin Index; name equivalent to THI for measurement of internal organs) when analyzing hyperspectral parameters during major hepatectomies after arterial vessel clamping. A strongly negative OHI value further correlated with more severe liver damage and postoperative liver failure [29]. In turn, Mehdorn et al. found significant StO_2_ reduction in acute mesenteric infarcts in the area of ischemic bowel segments, although the THI index did not show significant changes, which is in accordance to our results of dynamic skin perfusion [15, 30].

One major factor in the limitations of this study is the variability in light exposure during hyperspectral image acquisition, which could have led to inconsistencies to the resulting spectral data set and, consequently, in the calculated perfusion parameters. This issue is compounded by inter-operator variability in the application of local anesthesia, which might have introduced differences in administration and timing. Furthermore, a maximum measuring depth of 0.1 to 6 mm [31], anatomical differences among participants, such as variations in gingival thickness or vascular density, may have influenced the perfusion measurements, contributing to the observed variability in baseline values. Although statistical techniques such as use of randomization, correction of sphericity violations and repeated measures ANOVA were employed to minimize these influences, residual bias cannot conclusively be eliminated. To address these limitations and enhance the practical relevance of the results, future studies should validate eHSI in clinical settings with compromised patient populations, such as diabetes, vascular diseases, smoking habits, or other conditions affecting local tissue perfusion. Extending the observation period, akin to the approach taken by Thiem et al., may also yield more comprehensive insights into the correlation between measured values of StO_2_, NPI, THI, TWI, and their respective baseline values at various subsequent time intervals [15]. The present results enable promising future applications of eHSI, e.g. a more reliable assessment and success monitoring of periodontal therapies, microvascular flap perfusion monitoring, or non-invasive assessment of mucosal lesions with increased perfusion characteristics. The authors further see significant potential in Artificial Intelligence processing of hyperspectral raw data for the classification of premalignant precursor lesions or the evaluation of peritumoral mucosal regions for the individualization of surgical resection margins. By taking these factors into account in future research projects, the robustness of hyperspectral imaging as a diagnostic tool for intraoral perfusion monitoring can be further substantiated.

## Conclusion

The findings of the current study demonstrate for the first time the applicability of eHSI in assessing oral gingival blood flow, thereby laying the groundwork for various projects. StO2 and THI emerged as the most suitable HSI parameters for reliable detection of mucosal ischemia, showing strong correlations with clinically observed ischemic signs. Specifically, THI demonstrated a strong correlation (η = 0.7), accounting for 50.7% of ischemic changes, while StO2 showed a moderate correlation (η = 0.454), explaining 20.6% of the variance. Furthermore, the results indicate that after administration of ILA with 4% articaine and 1:200,000 epinephrine, the period of critical hypoxia does not exceed the threshold for irreversible damage, suggesting a low risk of papillary necrosis under these conditions.

## Data Availability

No datasets were generated or analysed during the current study.
